# GNSS Trajectory Anomaly Detection Using Similarity Comparison Methods for Pedestrian Navigation

**DOI:** 10.3390/s18093165

**Published:** 2018-09-19

**Authors:** Pekka Peltola, Jialin Xiao, Terry Moore, Antonio R. Jiménez, Fernando Seco

**Affiliations:** 1 Centre for Automation and Robotics (CAR), Spanish Council for Scientific Research (CSIC-UPM), Ctra. de Campo Real km 0,200, Arganda del Rey, 28500 Madrid, Spain; antonio.jimenez@csic.es (A.R.J.); fernando.seco@csic.es (F.S.); 2 Nottingham Geospatial Institute, The University of Nottingham, Triumph Road, Nottingham NG7 2TU, UK; jialin.xiao@nottingham.ac.uk (J.X.); terry.moore@nottingham.ac.uk (T.M.)

**Keywords:** similarity, GNSS trajectory, pedestrian dead reckoning, multipath, anomaly detection

## Abstract

The urban setting is a challenging environment for GNSS receivers. Multipath and other anomalies typically increase the positioning error of the receiver. Moreover, the error estimate of the position is often unreliable. In this study, we detect GNSS trajectory anomalies by using similarity comparison methods between a pedestrian dead reckoning trajectory, recorded using a foot-mounted inertial measurement unit, and the corresponding GNSS trajectory. During a normal walk, the foot-mounted inertial dead reckoning setup is trustworthy up to a few tens of meters. Thus, the differing GNSS trajectory can be detected using form similarity comparison methods. Of the eight tested methods, the Hausdorff distance (HD) and the accumulated distance difference (ADD) give slightly more consistent detection results compared to the rest.

## 1. Introduction

In urban environments, it is often the case that GNSS signals are blocked by either tall buildings or trees. Moreover, multipath makes it harder for a receiver to lock onto the affected satellite signal. GNSS signals affected by multipath or other anomalies are attenuated and do not arrive directly to the receiver, but are reflected from obstacles, such as buildings, and as a consequence, the measured pseudorange is altered from its correct value.

### 1.1. Research Objective

To compensate for unreliable GNSS positioning information, a foot-mounted dead reckoning system can be used to aid GNSS. An inertial measurement unit (Microstrain 3DM-GX4-45 and Xsens in our tests) was placed on the user’s foot. The pedestrian dead reckoning information from the IMU can be fused with the GNSS position measurements using, for example, a Kalman filter. The trouble is in defining how to weight the fusion of the unreliable GNSS positions with the dead reckoning information. The error estimate provided by the receiver is often unreliable, and using the receiver error estimate for fusion weighting does not always produce the correct track. In this paper, we develop a novel weighting method that assigns a weight to the GNSS position measurements depending on their estimated reliability (quality), before the fusion.

This paper focuses on examining the GNSS measurement quality derivation. A demonstrative example is shown towards the end of the paper that compares the fusion results between a simple weighting method against the novel weighting definition method. The novel weighting definition method compares the GNSS and Pedestrian Dead Reckoning (PDR) track shapes, in the GNSS measurement quality definition process. Before we look into the novel method developed in this research, we first need to become familiar with the basics of shape matching.

### 1.2. Defining Shape

What is shape? Form perception is one of the key abilities of the human mind in psychology. To achieve this, a human can rely on just a single aspect of an object, such as shape or size. Shape can be considered as a geometrical property defined by points, curves, surfaces, etc. To evaluate the similarity between two images, shape can be used as an important visual feature. Shape recognition pioneer, Mumford [1], studied shape recognition on Harvard students and pigeons.

Shape perception by humans is commonly based on the visual parts of an object. Latecki and Lakämper [2] used an example of shape recognition of the palm of the hand, to emphasize the phenomenon of recognizing the whole object (a person in this case) by only observing a single but significant visual part (hand). That is, by using any subshape of a larger shape, it is possible to apply a partial matching to capture subparts and regions. Compared to global matching, partial matching can be more complicated due to the related issues of scale selection and subpart selection. Latecki [2] aims to formalize shape representation and matching. He emphasizes that object-based geometric representation makes it possible to separate partial changes in the mapping environment. This is to contextualize objects or partition them into features.

Shape consists of features. To extract the shape from an image, several factors may influence the capturing, such as noise, defects, arbitrary distortion and occlusion. This implies that a shape can become corrupted easily. In [3], Grigorescu proposed a rich local shape descriptor by describing a 2D visual object based on different sets of feature points. He states that morphology is the study of form. Feature points and the distances between them are described as being perhaps the first features that come to mind when talking about a shape. By using a set of (labeled) distance sets, assisted by the feature points of an object, a two-dimensional (2D) visual object could be described. With an elaborated local descriptor, the salient features, which are also considered to be perceptually important features, including edge, corner, regions, or specific types of junctions at a given location in the image, can be captured. This shape describing information can be stored in data structures, as stated in [1].

Mumford [1] evaluated the performance of boundary curvature of a shape as a data structure for storing shape. As curvature can be considered invariable under rotation and translation, it can also be applied as an important perceptual feature. However, it could be highly unstable to reconstruct plane curves using only curvature.

### 1.3. Rigid Congruence

Latecki [2] divides shape descriptors into three categories: the contour, the area and the skeleton. The contour, in more detail, is based on the contour shape compared against known existing shape descriptors. The area method searches for a value inside the area defined by the shape points. The skeleton method simplifies the shape into a tree structure and compares these with a matching database. Of these, the contour-based method is stated to outperform the other two.

An important note is raised in [2], where they mention the cyclic ordering of the objects. This is where the objects (or the points or lines they consist of) are always defined in either a clockwise or counterclockwise manner. Shape description rules ease the matching process.

Bronstein [4] defines congruence, rigidity and isometry. Congruence retains the extrinsic geometry, which is the rigid motion (rotation, translation). In a rigid shape, the intrinsic and the extrinsic geometries are equal. An isometry preserves the intrinsic geometry (rotation, translation, reflection). In our work, we use rigid similarity of two trajectories. Or in other words, the dissimilarity between two position estimation tracks. Furthermore, we follow the process in [5], where geometric similarity consists of two phases, pose estimation and modification, and the similarity measurement. Ovsjanikovs [6] states that an m-dimensional Euclidean space has only m(m + 1)/2 degrees of freedom. In our case, 3 for the planar 2D track sections.

### 1.4. Similarity Measures

Similar feature comparisons between objects are exploited in many computer vision applications and matching problem solving. As [4] discusses, a similarity comparison asks a semantic question. Semantic, here, means the abstraction level of shape or the complexity of the shape. It can also be described as the relationship between two shapes’ features, like the similarity between the points, edges or lines. Simulating water and many natural characteristics, like fire, has improved, for example, in the animation film industry. Features can also be other than geometric, like, for example, skin tone, or the brightness of a picture. The application and the end user’s needs together define the semantics for the similarity criterion.

Similarity can be thought of as a variation difference between objects. Some objects have a basic and simple shape, while other objects are more complex and some are disturbed by noise. In both of the works [1,2], polygonal approximation was used. Latecki [2] presented a method where the shape was simplified by removing the irrelevant features first. They used a feature relevance classifier method to evaluate the importance of the features. Features can be, for example, corners found by corner detection methods or other clearly distinguishable properties in the object or image.

Objects can be partially hidden or occluded by other objects. Bronstein [4] used the fuzzified multicriterion and the Pareto distance for the partial shapes of one complete shape. Curvature characteristics can be compared partially, as well. The curvature detection is independent of the translation and rotation differences between two objects as [1] states.

Novotni [5] notes that the standard pose is often derived before comparison can be made. This is to remove the translation and rotation differences before the comparison. In [1,4,6,7], the authors mention the iterative closest point (ICP) distance method as the basic method for similarity comparison. This is stated to be a measure of incongruence. Furthermore, Hausdorff distance, Gromov Hausdorff for isometries with different reference metrics, templates, area and volumetric measures, energy, and partial matching are mentioned.

### 1.5. Application to Pedestrian Dead Reckoning and GNSS Tracks Comparison

Map matching is very often used in navigation applications. Latecki [2] uses shape recognition in matching localization information with mapping information. In this work, we use matching methods in the context of pedestrian navigation and for GNSS anomaly detection purposes by comparing the recorded points in the PDR and the GNSS tracks.

In [8], the sensors have different biases and error behavior, and in our case, we trust (the drift to be under 5%) the PDR solution. Although our PDR setup had its characteristic error tendency of turning slightly right, and we compare the GNSS multipath anomalies against this PDR track. We examine real GNSS anomaly/multipath examples and create simulated anomalies based on these. The simulated GNSS tracks with anomalies are then compared against a default PDR track that is presented later, using the methods introduced in Section 2.

The application of this comparison filter helps to fuse the GNSS measurement more correctly (with more successful weighting) with the pedestrian dead reckoning track. The prerequisite is the trust that in all situations, the PDR system is able to follow user motion within the error limits of 5% of the walked distance. A PDR quality system inspection needs to be developed to be able to create a more robust implementation of the comparison method. Then, the method described in this paper would be dependent on this PDR track quality measure, as well. For now, we trust the PDR track, as this functions for a normal paced walk and for the tests conducted in this work. For shuffling of feet and rapid movements, the method is not yet applicable without the development of a PDR track quality inspection system.

## 2. The Challenge and the Detection Methods

Magnetic storms, the differing ionosphere, the total electron content in the atmosphere and the weather, blocking trees and buildings, and multipath are a few examples of causes of anomalies in GNSS receiver position estimations. These changing errors need to be estimated and compensated for to get the most accurate results.

**Figure 1:**
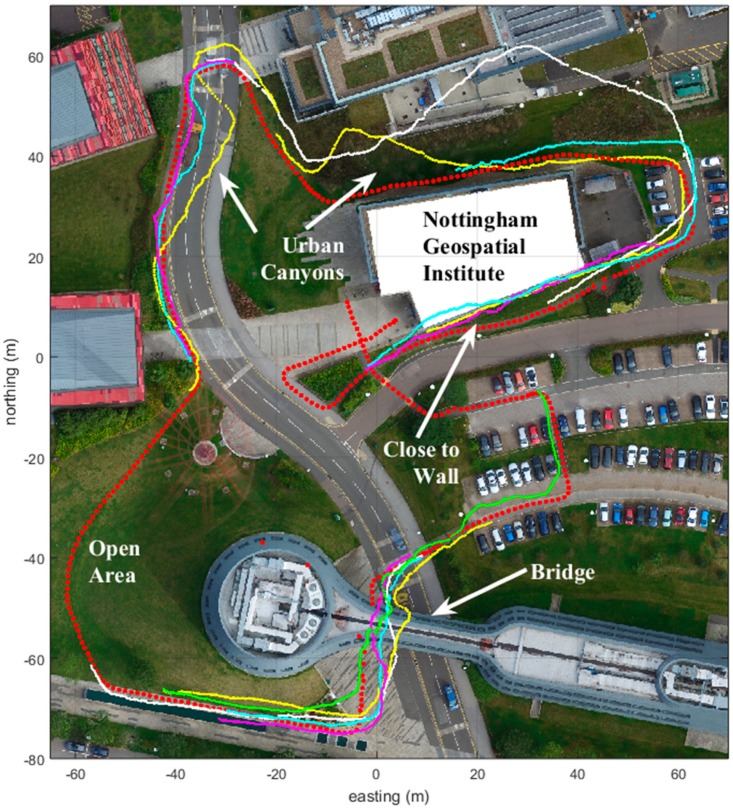
The red track shows the ground truth recorded using the Leica RTK kit. Other colors depict the characteristics of the anomalies in the GPS position estimations using u-Blox6 in the Microstrain 3DM-GX4-45 sensor. Measurements were taken on 23rd of September, 2016 between 11:00 and 12:00.

**Figure 2:**
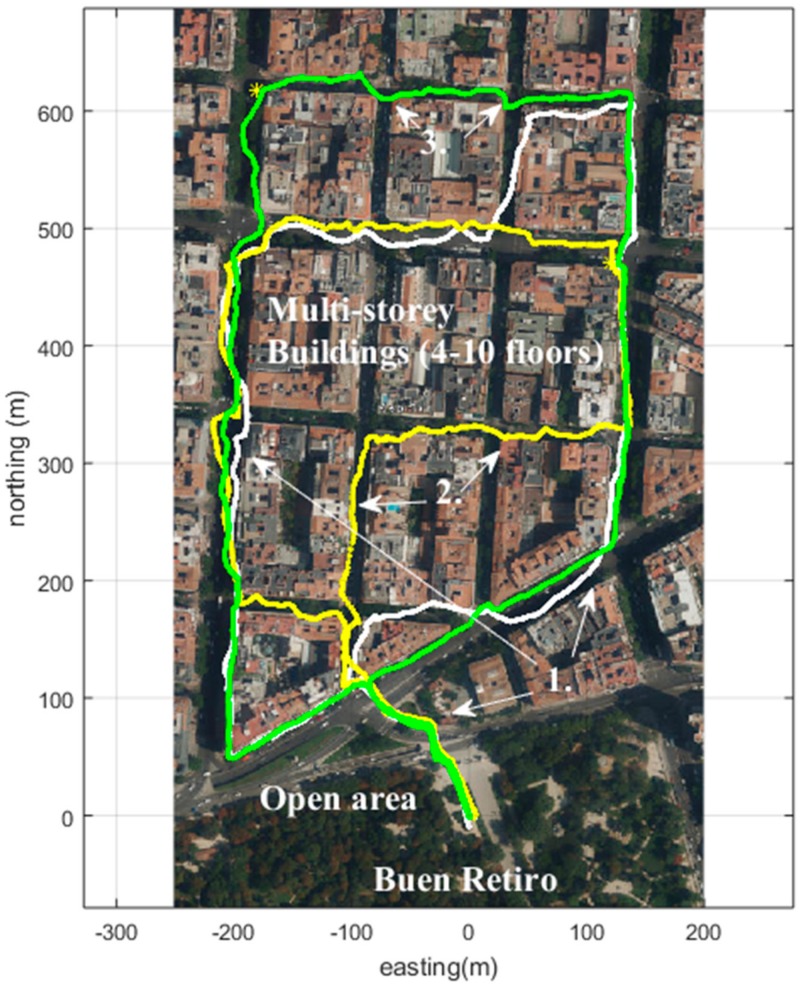
The green GNSS track was recorded 11:30 on 29th of August, 2018 using Samsung Galaxy S4 (GPS and GLONASS). The white track was recorded at 12:00. The yellow track was recorded at 12:30. Open area shows error below 4 m (1.). In the urban area in Madrid, with buildings, the error is greater (2.). In crossings the trail is affected (3.).

An example of improving the quality of the position measurement is mentioned by Pullen [9], called the geometry screening method, which is provided by the LAAS Ground Facility (LGF), for selecting trustworthy satellite geometries to be used by aircrafts. For pedestrians, and generally in the GNSS receivers used by the public, the quality information of the position measurements is often not trustworthy. This is especially true in challenging urban environments, where tall buildings affect the GNSS receiver position acquisition. Thus, the position information and the position error estimation information provided by the receiver are often erroneous. In this paper, we provide a novel way to derive the GNSS measurement quality information through a comparison of the pedestrian dead reckoning (PDR) and the GNSS track forms. The pedestrian dead reckoning track that is used in this paper was recorded using the Microstrain 3DM-GX4-45 sensor, feeding the gyro and accelerometer values to the 15-error-state Kalman filter. Figure 1 can be considered to have been recorded in a semi-urban area, where the GNSS signal is obstructed for up to approximately 80 m (depending on the available satellites and their locations), but not longer. An Xsens sensor was used to record PDR alongside the GNSS tracks of Figure 2, but the PDR trails were not used, since the dense urban environment needs more research.

Firstly, in this section, we examine the real-life GNSS trajectory anomalies of a pedestrian, walking in an urban setting. Then we create a random multipath generation procedure that creates multiple simulated examples resembling these real-life trajectories. Afterwards, we present the methods used in the comparison process of the PDR and the GNSS tracks for detecting the GNSS anomalies using the shape similarity techniques.

### 2.1. Recorded Anomalies and the Multipath Character

Figure 1 and Figure 2 show the collected examples with anomalies for the real GNSS trajectories. The used receiver was the u-Blox6 (GPS only) embedded in the Microstrain 3DM-GX-45 with a patch antenna. The red trajectory is the reference trajectory recorded using the Leica RTK kit. The rest of the colored tracks are used to indicate the anomalies at distinctive places in the multiple recorded GNSS trails.

Another test was made using the GNSS receiver in the Samsung Galaxy S4 mobile phone at the North side of the Retiro Park in Madrid. The resulting tracks are shown in Figure 2.

We see four specific areas in Figure 1 where the GNSS track behaves differently. In the southwest corner, no critical GNSS anomalies are present. This is an open outdoor area with good sky visibility. On the north side of the area there are four buildings that affect the GNSS receiver function. One of them is the Nottingham Geospatial Institute building.

We note the anomalies that make the estimated trajectory slowly drift away from the ground truth. In the east side of Figure 1 (Northing 40, Easting −40) and around the Nottingham Geospatial Building, the trail is affected. On the south side of Figure 1 (Northing −70, Easting 0), the user walks under the bridge, and this creates anomalies in the GNSS trails.

In Figure 1, we can see different types of anomalies for the u-Blox receiver-patch antenna combination of the Microstrain 3DM-GX4-45 sensor. The most common is a signal that slowly drifts away from the ground truth, and then slowly returns. We can also see a few more fast-returning signals, like the two yellow trails in the north side of Figure 1. The walk direction for the yellow signal, just to the north side of the NGI–building (Northing 40, Easting 0) is from the east to the west (counter-clockwise). Other trails are clockwise walks. In addition, a slowly drifting, zigzag-style disturbance can be detected. The typical length is from 10 m to 100 m, and the typical width is generally around 5 m, but with deviations of up to 30 m.

In Figure 2, the mobile phone GNSS receiver using GPS and GLONASS satellites was used. The errors in the urban canyons resemble the errors in Figure 1, with deviations of up to 30 m. We can see that in the open area (Number 1, and on the south side of Figure 2), the positioning error is below 4 m, and we can distinguish on which side of the street we are walking. However, when entering the area with buildings 4 to 10 levels tall, this is no longer true; we can determine the road on which we are located, but not the correct side. It can be noted that on the easternmost street, compared to the westernmost (see Number 1 arrows) street, the error is smaller. We do not analyze it here, but for the curious, at the measurement times noted in Figure 1 and Figure 2, the skyplots with locations of the satellites in the sky can be seen in, for example, (http://gnssmissionplanning.com/App/Skyplot).

In Figure 2, the usual positioning result in the urban area with buildings is shown with the Number 2 and arrows. The result sways between both sides of the street and makes it impossible to say on which side of the street the user is. Also, often, when coming to street crossings, the positioning result trail has a noticeable bump, although walking straight ahead (Number 3).

### 2.2. The Simulated Anomalies and the Generated Multipath

A MATLAB GNSS multipath generator code was used to create random multipath-style deviation for a straight-line trajectory. Three types of multipath were randomly generated, named basic, abrupt and zigzag multipath. The basic multipath first randomly picks the side, left or right, onto which side the multipath will be added. Then a width is randomly drawn that is a number between 0 and 30 m, weighted to select more deviations of approximately 5 to 10 m. This defines the maximum deviation of the multipath to either side. A guided and smoothed random walk is then allowed to reach this limit and return back to 0. The random walk characteristic drift can be changed. For the basic option it is relatively slow. The length of the basic multipath is randomly drawn from between 10 and 80 m, favoring lengths of around 20 to 30 m.

The abrupt multipath is similar to the basic, until the width is reached using the random walk function. The return to 0 is more abrupt, whereby the random walk characteristic settings generate a steeper return.

**Figure 3:**
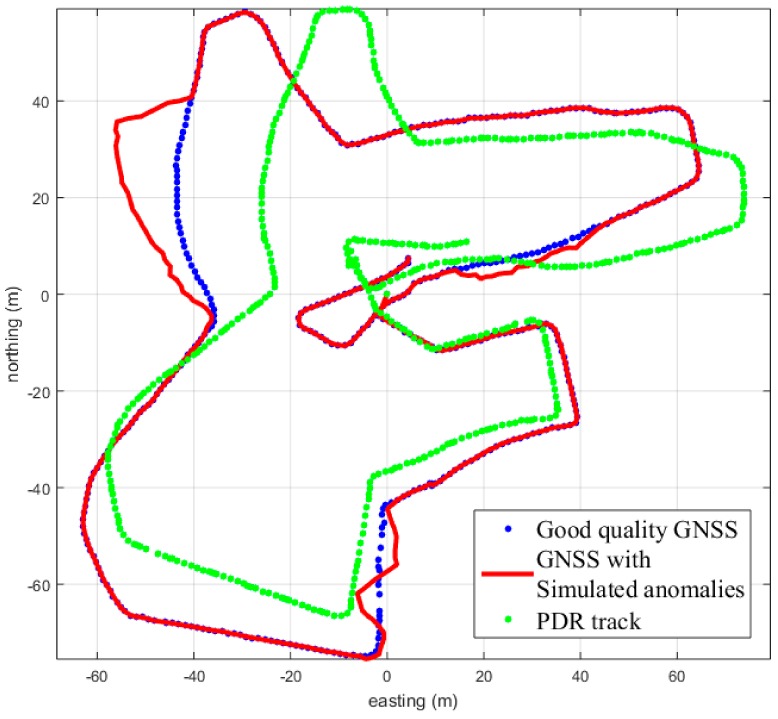
The red track shows the added simulated GNSS trajectories. The blue one is the original good-quality GNSS track. The green track shows the PDR trajectory, which is compared with the GNSS tracks with anomalies.

The zigzag multipath width is between 0 and 20 m for both sides. Slightly faster random walk characteristics are used compared to the basic settings. The zigzag multipath first reaches the other side, a randomly drawn maximum, before turning to drift the defined amount in the other direction. The number of zigzag turns is a random number between 2 and 5. Typical simulation results are shown in Figure 3 below.

Three typical points were selected, around which three multipath additions were attached. These are just before the bridge around the coordinates Northing −40 and Easting 10; in the urban canyon, Northing 20, Easting −40; and close to the NGI wall at Northing 30, Easting 60. Figure 3 shows the good-quality GNSS and the PDR tracks in blue and green, respectively. The red track shows an example of the added simulated anomalies to the good-quality GNSS trail.

The PDR track is compared against the GNSS tracks with the simulated anomalies. The southernmost anomaly in the example of Figure 3 is of the zigzag type, the western anomaly is abrupt, while the eastern anomaly is of the basic type. The generation weighted the appearance of the anomalies so that the basic type was the most common and appeared over 70% of the simulated multipath. Then, the abrupt, with 20% probability, and 10% probability for the zigzag type to be placed onto the three slots on the complete GNSS track. In the same way as the length of the multipath track, the starting point of the multipath was also randomized to start within limits, so that the anomalies wouldn’t overlap with each other. Next, we introduce the methods that were used to compare the two tracks: the good-quality PDR and the GNSS with added anomalies tracks.

### 2.3. Preprocessing and Similarity Comparison Methods

To detect the anomalies and to conduct the comparison process between the track sections on the PDR and the GNSS tracks, the pose problem first needs to be solved. Similarly, as explained in [5], we first tackle the pose problem. This means that the translation and rotation differences of the track sections are eliminated.

The starting points of each track section under investigation are first moved to zero. Then, the end points are rotated to the positive *x*-axis. After this, the GNSS track sections with the anomalies and the PDR track differences can be evaluated using all of the following methods and the method efficiencies compared.

#### 2.3.1. Turning Function Distance, TFD

The Turning Function Distance is introduced in [10]. This method is invariant to the translation difference between the two tracks if we can define the starting point of the function to be the same on the two trails that will be compared. In our case, this is true, since we pick the same portions of both the GNSS and the PDR trails.

The cumulative angle function is run through both tracks. Left-hand turns increase the accumulated angle and right turns decrease the angle. The distance is measured while advancing forward along the shape. The total distance can be normalized in order to compare just the turns and at which points of the shape the turns happen. The cumulative angles on both of the tracks can then be compared. A dissimilarity measure can be derived from the distance difference between the two cumulative angles. This is the Turning Function Distance:

(1) $$d\left(A,B\right)={\left[\int {\left|\theta_{A}\left(s\right)-\theta_{B}\left(s\right)\right|}_{p}\cdot ds\right]}^{\frac{1}{p}},$$

where *θ* is the accumulated angle on track *A* and *B*, *s* is the arc length travelled and p is the dimension.

A threshold is used to indicate a detection of an anomaly in the GNSS track if this distance gets too big. The threshold was set using a training set of 100 simulated GNSS anomalies tracks, so that the anomaly detection percentage was searched and set highest for the training set. Here, the GNSS track sections with known anomalies were compared against the good-quality PDR trail. Deriving the thresholds using the ideal GNSS trail would be preferred, but for this work, we used a PDR trail that tends to turn to the right.

#### 2.3.2. 3-Point Angle Difference, 3PAD

**Figure 4:**
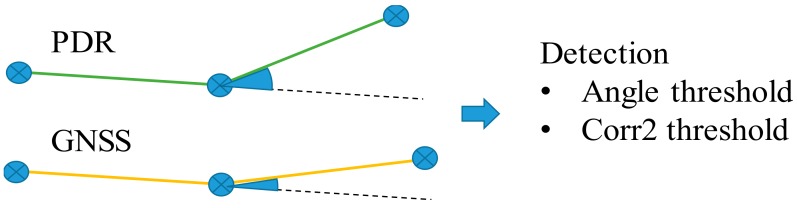
Three points on the PDR and GNSS tracks are taken, with intervals of 5, 10 and 15 m. The angle between the two lines formed by the three points is derived for both tracks. An angle threshold then classifies the GNSS track section as being of good or bad quality. Another method uses the three points and feeds them to the corr2 function in MATLAB.

A simple three-point angle difference method was also examined. We picked the three points at 5 m, 10 m and 15 m intervals. Here, the start point and the end point with a point in between are taken, and the angle between the two lines formed by the three consequent points is derived for both tracks. Again, if crossing the angle threshold, this will indicate an anomaly on the GNSS track. Figure 4 depicts the angle difference. The threshold was determined that maximized the anomaly detection rate for the simulated training set.

#### 2.3.3. 3-Point Correlation, 3PC

Furthermore, the correlation is calculated for the three points on both tracks. This is implemented using the corr2 function in MATLAB. The corr2 output calculates the relative pixel intensities for two images, or in our case, the two trails. A threshold value indicates the classification between similar or not similar trails. This threshold was again derived using the training set. As a side note, blurring the trails could be a novel idea for the further testing of this correlation method, but we did not test it here due to lack of time. Figure 4 shows the three points on both tracks.

#### 2.3.4. Multi-Point Correlation, MPC

In addition, the correlation using all of the points in the track sections under investigation were input into the corr2 MATLAB function. Again, a threshold derived using the training set separates the two cases, the anomaly detected and the no anomaly detected.

#### 2.3.5. Area Difference, AD

**Figure 5:**
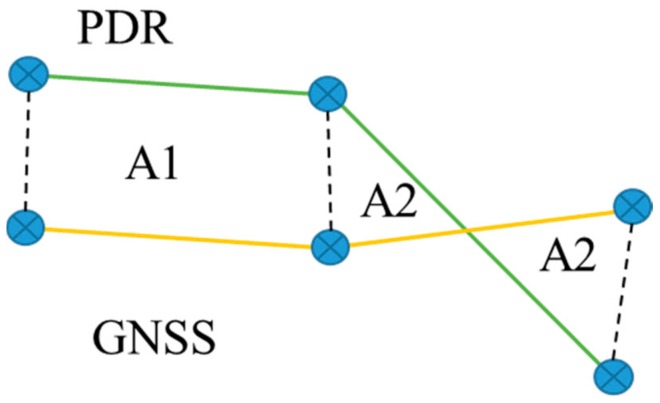
The trapezium area is calculated as defined between the PDR and the GNSS tracks. A threshold of the total area along the track section under investigation classifies the detection.

The area between the PDR and GNSS tracks is calculated using the trapezium area calculation methods. Most of the time the area of a quadrilateral is calculated between the consequent points on both tracks. When the two tracks cross each other, the cross-quadrilateral area is calculated. An area threshold detects the anomalies, and this threshold was determined again using the training set for the maximum detection percentage of anomalies. Figure 5 describes the area calculation method.

#### 2.3.6. Accumulated Distance Difference, ADD

The point-wise accumulated Euclidean distance difference is calculated between the two track sections, the GNSS trail with anomalies and the PDR trail. This distance difference is summed over the complete investigated track section. A threshold value is used to indicate the anomaly detection. The threshold is again determined by using the training set. The training set consists of the 100 simulated GNSS tracks with anomalies and is compared against the PDR trail shown in Figure 3.

#### 2.3.7. Hausdorff Distance, HD

The Hausdorff distance measures how far two sets of points are from being isometric. The Hausdorff Distance method finds the maximum of the two directional distance searches between point groups A and B, in this case, the points of the two trails. The two directional searches find the point that is furthest from any point in the other set. Then, the resulting Hausdorff distance of the two directional searches is the maximum distance of the two points from their nearest neighbors in the other set.

d(A,B) = max{directional_search(A,B),directional_search(B,A)}(2)

Again, the threshold is determined using the training set, and a value is chosen such that it produces the maximum detection for the training set.

#### 2.3.8. Modified Hausdorff Distance, MHD

The modified Hausdorff distance is explained in [11]. This approach is more complex, and includes 6 types of directed searches that can then be combined into 4 further undirected distance measures. This creates 24 options of distance comparison values, of which one is the Hausdorff distance. Appropriate thresholds were used to classify the detection outcome derived by the training set.

## 3. Results

The comparison setup consisted of a combination of three different-sized detectors with lengths of 10, 20 and 30 m. The detection percentages in four cases were examined. Separately, using the three detectors, and also using the OR combination of the three detectors. That is, if any of the detectors detects an anomaly, then the anomaly is detected. The best thresholds that produced the greatest detection percentage were determined using the training set for each separate detector (10 m, 20 m and 30 m). The OR combination used these best settings.

**Figure 6:**
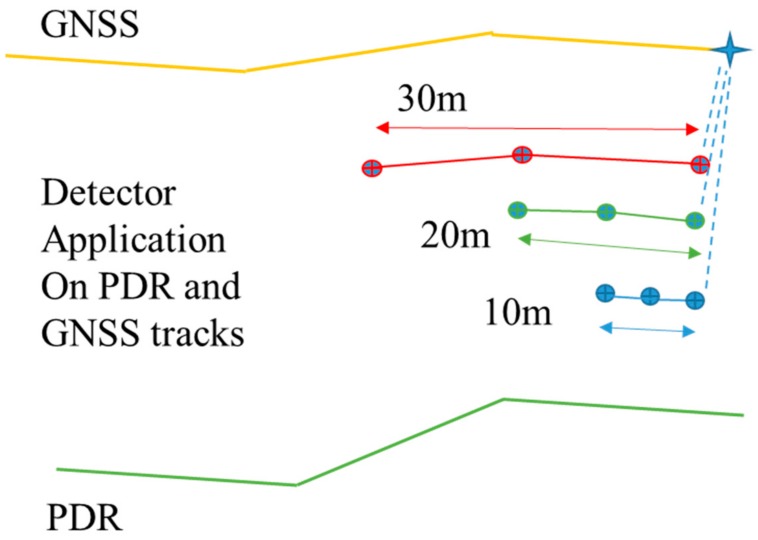
The three detectors are applied so that the first points are at the current measurement.

The PDR track was trusted to track the position change up to 30 m. Then, each of the methods in the previous section was applied using the three different-sized detectors. The three sizes were track sections of 10 m, 20 m and 30 m. Figure 6 depicts how the filters were implemented and processed. The track sections to be compared start at the same current measurement position. The three different-sized detectors are used on both tracks, the PDR and GNSS trails and the points (the 3 points shown in Figure 6) included in these track sections are fed to the detection methods.

An anomaly is detected if any of these different-sized filters returns a positive detection. At first, we examine the detection accuracy of the different methods. Afterwards, we consider the computational speed and the characteristics of the detectors.

### 3.1. Detection Accuracy

One hundred tracks were generated using the developed anomaly simulation code, each consisting of three multipath anomaly sections. A successful detection is defined in the following manner:

“Detection is successful if within the track section in question there is a simulated multipath point and the detector returns a positive detection. Also, detection is successful if within the track section in question there are no simulated multipath points and the detector returns a negative detection. Otherwise the detector fails to indicate correctly. The success is marked at the current measurement and compared with the ground truth.”

A detection percentage then indicates the filter efficiency in terms of detection accuracy. Table 1 summarizes the final detection accuracies for the different methods. The OR combination of the 3PAD and ADD detectors was added after their individual characteristic behavior was analyzed. If there is any detection by at least one detector, a detection is registered. The individual behavior and feature examination follow in the Discussion section.

### 3.2. Computational Efficiency

Table 2 summarizes the computational speed relative to the fastest method, which is the 3-point angle difference (3PAD) method.

## 4. Discussion

The OR function is the primary detector that we focus on comparing now. The turning function distance TFD performed the worst. In particular, the longer interval track detector couldn’t achieve detection accuracies greater than 76%. For the TFD, this resulted in the poor result of the OR function as well, which indicates that the 15 m interval detector is very sensitive for indicating bad-quality GNSS measurements. This was somewhat expected, since for the longer track sections, the accumulated angle difference tends to get bigger, even with nearly identical tracks. Thus, this method is not very suitable for the detection process because of its sensitivity to small fluctuations in both the PDR and the GNSS tracks. These fluctuations are independent for both tracks and cannot be prevented from affecting the TFD detection. Loosening the 10 m and 15 m interval filter thresholds, the combined OR result improves by 2% to 77%. A further option could be to simplify the two tracks, but this would mean that we lose information of the two tracks, which we cannot do since it is essential information because we are trying to detect these anomalies, not to discard them.

The most accurate detectors are the Area Difference AD and the Modified Hausdorff Distance MHD detectors. However, these are also very much slower than the other methods; thus, using them for detection would require more attention to the overall end application computation resource design. An approximation of the area method could make the method faster, since the detection of two lines crossing takes most of the computation time. The 3PAD and ADD detectors are not much less accurate than these. Thus, with regard to detection accuracy, the 3PAD, ADD, and their combination, the correlation methods 3PC and MPC, and the HD method perform nearly equally. The detection accuracy in number is not, then, the decisive factor. The decisive factor in our test cases was the characteristic differences in performance between the methods.

### 4.1. Detection Methods’ Characteristics

**Figure 7:**
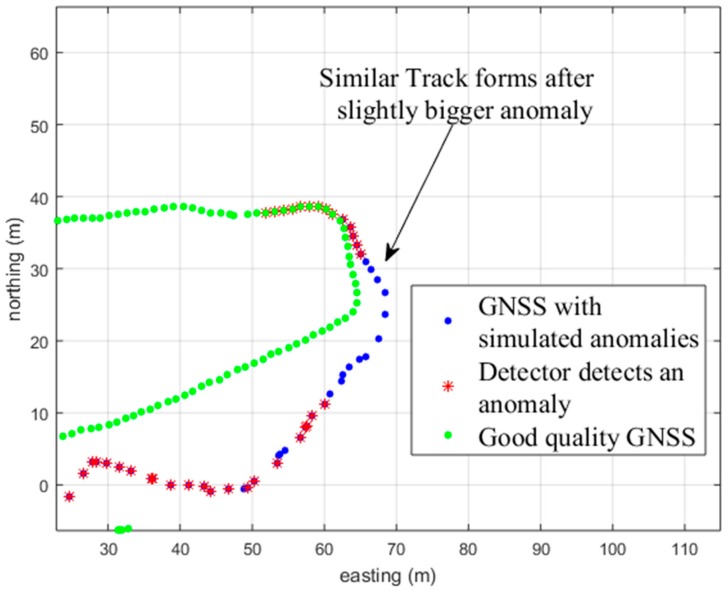
The green track shows the original good-quality GNSS track. The blue track is the GNSS track with the simulated anomalies, and the red bullets indicate a detection by the method used (3PAD).

With the 3PAD method, a drawback is shown in Figure 7. The 3PAD method compares the angle differences for the three different intervals. If there is a track section, like in Figure 7, where the anomaly is clearly a distinctive angle change at the beginning of the anomaly, and throughout the anomaly the angle changes are otherwise similar, the detector is unable to indicate these differences.

**Figure 8:**
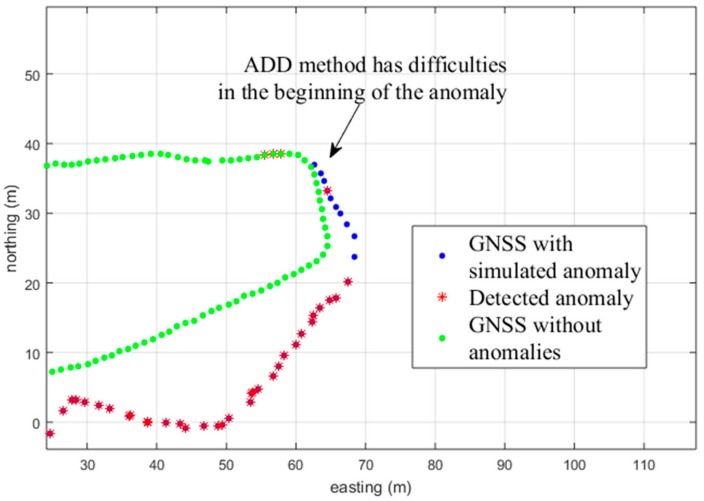
The green track shows the original good-quality GNSS track. The blue track is the GNSS track with the simulated anomalies and the red bullets indicate a detection by the method used (ADD).

Figure 8 shows the ADD method result for the same track section. Here is a typical drawback for the ADD (and HD and MHD) method. In the beginning, the accumulated distance is not large enough to cross the threshold value. Thus, the detection result in the beginning is erroneous. The OR combination of ADD with the 3PAD method improves resistance to these errors, since the first erroneous angle is detected by the 3PAD method.

**Figure 9:**
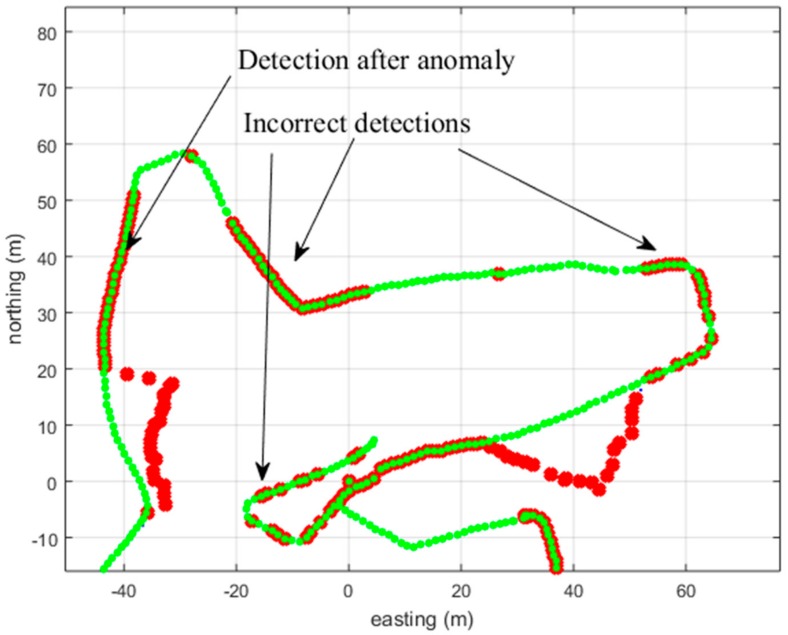
The green track shows the original good-quality GNSS track. The blue track is the GNSS track with the simulated anomalies (this is not seen because of the red detection markers) and the red marks indicate a detection by the method used (TFD). The TFD method, especially the long interval implementation, tends to indicate the track as being of poor quality (with incorrect detections shown). The drawback remains even when threshold values are loosened.

The TFD method had a strong tendency, and the 3PAD a moderate tendency, to indicate three turns (N30, E-10; N40, E60; N0, E-15 in Figure 9) as being of bad-quality GNSS, although in reality they were not. The false detection can be seen in Figure 9 for the TFD. The detection of these turns was moderately wrong with the correlation methods 3PC and MPC and for the combined 3PAD/ADD. For the AD and the MHD methods, this was mildly wrong, and for the ADD and HD, it was very mildly wrong. The strong tendency of the TFD method is shown in Figure 9 (incorrect detections), while the very mild tendency corresponds to none or only a few (1–5) red points appearing in these turns for the ADD and HD detection methods.

Each of the methods had the same effect as is shown on the left side of Figure 9, wherein, after the anomaly, the GNSS track is detected as being of bad quality even though it is not. This is mostly due to our detector application at the current measurement point, as shown in Figure 6. Different application locations on the track (defining the quality of the middle point or the oldest point in time instead of the current time) could improve the detection accuracy and make the detector more usable.

### 4.2. Applying the Detection Method

**Figure 10:**
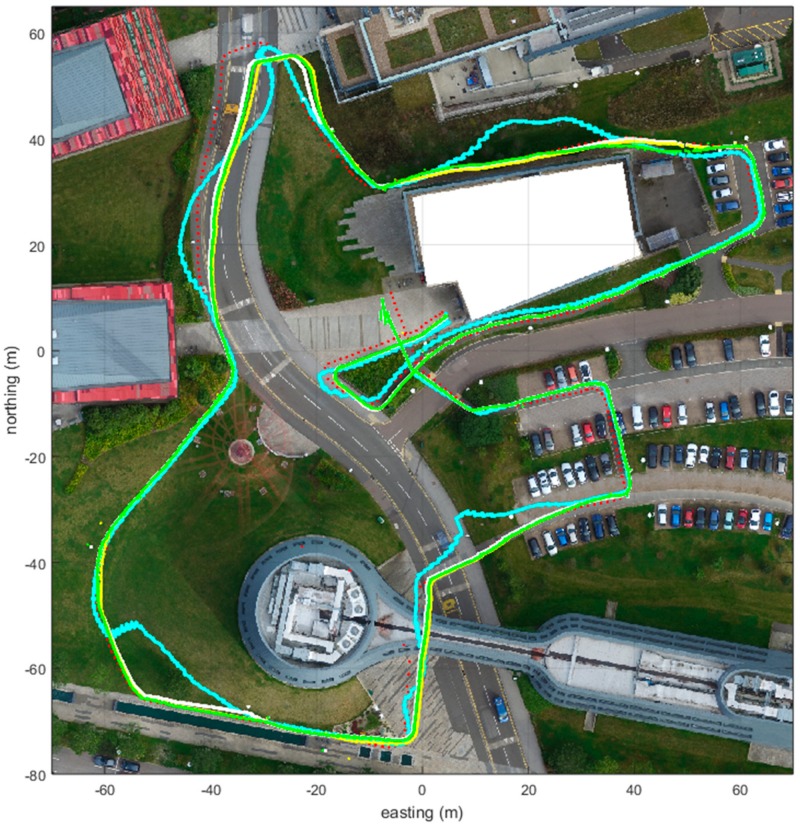
When the anomaly detection method is not used and the GNSS measurements are treated as being of equal quality, the resulting fusion of the PDR and the GNSS with the anomalous trails produce the cyan track, which follows the anomalies. Use of the GNSS anomaly detection method the GNSS measurements can be fused so that the GNSS measurements with anomalies are weighted much less, and the trail resembles the true trajectory (white, green and yellow tracks resemble the red track form, which is the ground truth).

When we apply the detection methods to a simulated case, we can see the benefit of the method in Figure 10 (3PAD/white, ADD/yellow and HD/green in this example case). Without using the novel similarity detection method, the PDR + GNSS fusion using the equal-quality GNSS measurements also follows the anomalies, as shown with the cyan color in Figure 10.

Instead, when applying a low-weighted real-time fusion for each GNSS measurement, and then after the detection method application, a delayed heavy-weighted GNSS measurement fusion for the good-quality tagged GNSS measurements, the fusion is more successful and is able to construct a trajectory closer to truth. The accuracy is improved by a factor of approximately 1.72 for all three detector methods in the example case. The longer the anomaly is, the harder it is to compensate for (e.g., anomaly at N40, E−40). The delayed track form match GNSS fusion is further explained in [12].

## 5. Conclusions

Considering the results and the discussion section, the detection percentages are similar for all the detectors, except for the TFD, which is somewhat worse. The decisive factors are thus not the detection accuracy but the specific features and the computational speed of the detectors. It should also be noted that the multipath severity differs. A multipath anomaly that sways (zigzags) near the ground truth is not as critical as a multipath that shows the resulting position to be far away. In our tests, the severity was not taken into account. A manual analysis was conducted to visually inspect the detection results for the 100 tracks for each method, revealing the characteristics discussed in the Discussion section.

In conclusion, the HD or ADD methods are the preferred methods among those tested for the tested simulation examples. That is because these two methods do not have as much tendency to falsely mark the described track corners as bad quality GNSS. The quality of the PDR track plays a part in this, but it is the same for each tested method. Although the HD and ADD methods have a similar detrimental tendency at the beginning of the anomaly to mis-detect poor-quality GNSS measurements, as shown in Figure 8, still, their speed, together with their better trajectory corner detection (Figure 9) makes these two methods preferable. In addition, in the fusion application guided by the detection method, it is preferable that the detection method does not mark additional false track sections. In other words, the detection accuracy result is not as important as marking, at least adequately, the correct track sections. Combination with the 3PAD method could enhance the behavior of the detection filter, especially for the case demonstrated in Figure 8, to be able to detect early deviation, as shown in Figure 7 using the 3PAD method. However, this needs more testing, since the 3PAD method has a moderate tendency to falsely detect the corners, as shown in Figure 9 for TFD.

The purpose of the detection method is to help in defining the weight of the GNSS measurement for the fusion with the PDR track. We used detection methods indicating the quality of the current measurement. A better approach would possibly be to use the detectors defining the quality for the furthest point (30 m), instead of the current point, or for the middle point. A low-weight fusion in real-time and a delayed correction update according to the similarity detector result help in fusing the GNSS measurements more closely to the true quality of the GNSS measurements. In other words, this can be described as trusting the PDR solution for 30 m, and then, by form matching, fixing and fitting the PDR track to follow the good-quality GNSS measurements. This approach would be better for the fusion of the GNSS measurements after the track forms are compared, rather than at the current measurement or using questionable receiver error estimates directly. This approach is related to the track-to-track matching association investigated in [8].

It should also be noted that this method was developed for the situation of short GNSS gaps and short periods of bad quality measurements, such as in Figure 1. This means low-quality GNSS track sections that are less than 80 m. For example, for the case in Figure 2, the duration of the bad-quality GNSS measurements spans almost 1 km in distance. For these situations, when an absolute position cannot be guaranteed, a map matching or other form of additional assistance is necessary.

## Figures and Tables

**Table 1:** The detection accuracies of each detector separately and for the OR combination.

Methods	Detection Accuracy for the 5 m Interval Detector	Detection Accuracy for the 10 m Interval Detector	Detection Accuracy for the 15 m Interval Detector	Detection Accuracy for the OR Combination
TFD	83%	79%	76%	75% (77%)
3PAD	82%	82%	78%	84%
3PC	82%	83%	80%	83%
MPC	85%	88%	85%	83%
AD	85%	86%	82%	85%
ADD	82%	82%	78%	82%
HD	82%	82%	80%	81%
MHD	86%	86%	82%	85%
3PAD + ADD	85%	84%	82%	83%

**Table 2:** The speed of the computation for the different filters compared against the fastest, the 3PAD method.

Methods	The Relative Speed Against the 3PAD
TFD	5.5
3PAD	1
3PC	3.9
MPC	3.8
AD	152
ADD	2.4
HD	4
MHD	40
3PAD + ADD	3.4
